# Hyaluronic Acid Treatment Improves Healing of the Tenorrhaphy Site by Suppressing Adhesions through Extracellular Matrix Remodeling in a Rat Model

**DOI:** 10.3390/polym13060928

**Published:** 2021-03-17

**Authors:** Kwang Hyeon Ahn, Eun Soo Park, Chang Yong Choi, Han Gyu Cha, Yongsung Hwang, Seung Min Nam

**Affiliations:** 1 Department of Plastic and Reconstructive Surgery, Soonchunhyang University College of Medicine, Soonchunhyang University Bucheon Hospital, Bucheon 14584, Korea; 124874@schmc.ac.kr (K.H.A.); peunsoo@schmc.ac.kr (E.S.P.); 73120@schmc.ac.kr (C.Y.C.); prs.cha@schmc.ac.kr (H.G.C.); 2 Department of Integrated Biomedical Science, Soonchunhyang University, Asan-si 31538, Korea; 3 Soonchunhyang Institute of Medi-Bio Science (SIMS), Soonchunhyang University, Cheonan-si 31151, Korea

**Keywords:** tendon injury, tendon adhesion, hyaluronic acid, transforming growth factor-β1, plasminogen activator inhibitor-1

## Abstract

Due to the limited supply of vessels and nerves, acute or chronic tendon injuries often result in significant and persistent complications, such as pain and sprains, as well as the loss of joint functions. Among these complications, tendon adhesions within the surrounding soft tissue have been shown to significantly impair the range of motion. In this study, to elucidate the effects of a hyaluronic acid (HA) injection at the site of tenorrhaphy on tendon adhesion formation, we used a full transection model of a rat’s Achilles tendon to investigate the anti-adhesive function of HA. Our initial findings showed that significantly lower adhesion scores were observed in the HA-treated experimental group than in the normal saline-treated control group, as determined by macroscopic and histological evaluations. Hematoxylin and eosin, as well as picrosirius red staining, showed denser and irregular collagen fibers, with the larger number of infiltrating inflammatory cells in the control group indicating severe adhesion formation. Furthermore, we observed that the expression of tendon adhesion markers in operated tendon tissue, such as collagen type I, transforming growth factor-β1, and plasminogen activator inhibitor-1, was suppressed at both the gene and protein levels following HA treatment. These results suggest that HA injections could reduce tendon adhesion formation by significantly ameliorating inflammatory-associated reactions.

## 1. Introduction

Due to the limited supply of vessels and nerves, acute or chronic tendon injuries of the hand often result in significant pain and sprains, as well as the loss of joint mobility and stability in patients [1,2]. The most significant and persistent complication of these injuries is tendon adhesion, which leads to problems in joint flexion and contracture, occurring in approximately 30–40% of both repaired and unrepaired cases [3,4]. Numerous clinical cases and basic experimental reports suggest that the formation of tendon adhesions is mostly caused by inflammation of the injured tendons, surgical manipulation, and immobilization after surgery [5]. During tendon regeneration, fibroblasts in native tissues migrate from the surrounding tissue to the injured end of the tendon, leading to adhesion formation between the tendon and the surrounding tissue [6].

To overcome this severe post-operative complication, various attempts have been made to prevent tendon adhesion formation [7,8]. During the early stages of tendon regeneration, immobilizing the tendon tissue is critical for faster healing; however, at the same time, applying mechanical load onto the tendon tissue during the healing processes has also been shown to decrease the formation of post-operative tendon adhesions [9]. In addition, proper selection of the incision line and an appropriate choice of suture materials are known to be critical for preventing tendon adhesions [10]. Therefore, there has been tremendous interest in the development of anti-adhesive agents for the suppression of adhesion formation between tendons and the surrounding connective tissue [11].

Among the numerous anti-adhesive agents that have been developed to date—such as alginate, chitosan, carboxymethyl cellulose, gelatin, silk fibroin, collagen, and hyaluronic acid (HA) as natural polymer-based barriers, as well as polyethylene glycol, polylactic acid, polyvinyl alcohol, and poly ε-caprolactone as synthetic polymer-based membrane-like barriers, which have been shown to prevent tendon adhesions—many have exhibited trade-offs between biological functions as anti-adhesive materials and mechanical stability [4,12,13,14]. In particular, HA has been extensively investigated because of its unique biological properties and biomechanical nature [15]. In addition to lubricin, HA, a major component of the synovial fluid, is one of the biological fluids that exhibits lubricating capability. HA is secreted by the tendon sheath, plays a key role in smooth tendon gliding, and provides nutrition to the tendon [16,17]. Preliminary investigations have studied the effects of HA for the promotion of tendon healing and the prevention of post-operative tendon adhesion [18,19]. However, a limited number of studies have histologically evaluated adhesion sites following the use of HA as an anti-adhesive agent. Thus, in this study, we performed qualitative and quantitative analyses to assess the effects of HA in preventing tendon adhesion in the repaired Achilles tendon using a rat model. Histological characteristics and adhesion marker expression were compared between the HA-treated group and the control group, which was treated with normal saline (NS).

## 2. Materials and Methods

### 2.1. Animals

First, twenty four male Sprague–Dawley rats (5 weeks old, weighing approximately 200–250 g) were stabilized via administration of 1,4-Butanediol diglycidyl ether (HyFence^®^; Ildong Pharmaceuticals, Seoul, Korea) and then underwent Achilles tendon transection. The rats were then randomly assigned to the NS-injected control group (*n* = 12), hereafter referred to as the NS group, or to the HA-injected group (injection of 2% *w*/*v* HA; M_w_ = 708 kDa, Hyundai Bioland, Cheongju, Korea) (*n* = 12), hereafter referred to as the HA group. The ruptured tendon was repaired as per the modified Kessler method [20]. The tendon tissues were collected and analyzed 10 weeks after transection and repair surgery. All animal studies were approved by the Institutional Animal Care and Use Committee of Soonchunhyang University Bucheon Hospital (protocol number: SCHBC-A-2018-13).

### 2.2. Surgical Procedures

**Figure 1:**
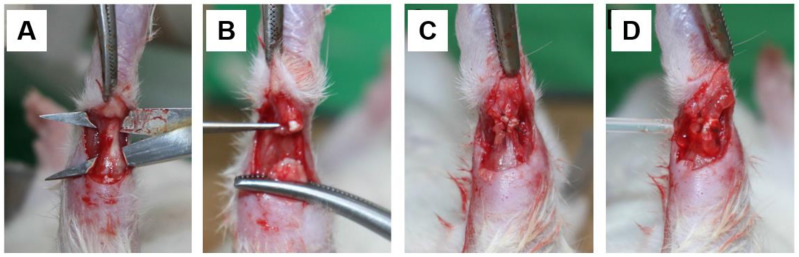
Graphical illustration. (**A**) The Achilles tendon is exposed. (**B**) A sharp incision is made with Metzenbaum scissors. (**C**) Primary tendon repair is performed following the modified Kessler method. (**D**) Hyaluronic acid (HyFence^®^; Ildong Pharmaceuticals, Seoul, Korea) is injected around the repaired tendon of the rats in the experimental group.

The operation was conducted under aseptic conditions. Animals were anesthetized via ketamine injection (100 mg/kg). The right hind limb of each rat was shaved and disinfected with alcohol and iodine. Using a posterior longitudinal incision with sharp dissection, the Achilles tendon was exposed and transected at the midpoint. The transected tendon was repaired using a modified Kessler suture with 6-0 nylon [20]. Before the closure of the wound, an intravenous cannula was placed at the tendon transection site to ensure that the injected solution (HA or NS) would fill the proper region. As a control, 0.3 mL of sterile NS solution was injected through the cannula, and subsequently, the skin incision was closed (Figure 1). In the same manner, an identical volume of sterile HA solution was administered to the rats in the HA group. External immobilization was not performed after surgery. The rats were fed standard chow, kept under a 12-h light/dark cycle, and had free access to water in their home cages.

### 2.3. Macroscopic Examination

At the observation time point, the rats were euthanized via high-dose ketamine injections, and the Achilles tendons were dissected along with the surrounding soft tissue. Through loupe magnification, the tendons were graded for adhesion formation in the repaired tendon, tendon sheath, and the surrounding soft tissue. Furthermore, the criteria described by Tang et al. for quantitative evaluation was used (Table 1) [21,22], and the corresponding tendon tissues were harvested and processed for microscopic evaluation.

### 2.4. Histological Evaluation

The tendon specimens were fixed in 4% *w*/*v* paraformaldehyde solution in phosphate-buffered saline (PBS) at room temperature. After the dehydration of the specimens using gradually increasing ethanol concentrations, samples were embedded in paraffin and excised into 5-µm-thick longitudinal sections. The sections were stained with hematoxylin and eosin (H&E) and washed with PBS. The stained slides were observed under a polarized light microscope. For H&E staining, we compared the mean number of inflammatory cells between the NS and HA groups. Picrosirius red staining was used to assess the differences in collagen distribution between the two groups using a commercial test kit (cat# ab150681, Abcam, Cambridge, UK), according to the manufacturer’s instructions. The number of collagen fibers in the images of high-power fields from each slide was quantified using ImageJ^®^ software (National Institutes of Health, Bethesda, MD, USA). All histological evaluations were performed from two random fields of view from twelve biological replicates per group at 10× magnification.

### 2.5. Immunohistochemistry

For immunohistochemical analysis, the slides were subjected to antigen retrieval in a microwave set to high power for 10 min, followed by immersion of the samples in hydrogen peroxide for 10 min. The sections were incubated with primary antibodies, including an anti-plasminogen activator inhibitor (PAI)-1 antibody (cat# ab66705, Abcam, Cambridge, UK) and an anti-transforming growth factor (TGF)-β1 antibody (cat# ab92486, Abcam, Cambridge, UK) overnight at 4 °C. Subsequently, the slides were incubated with a horseradish peroxidase-conjugated secondary antibody (cat# ab236469, Abcam, Cambridge, UK) for 15 min at room temperature, developed with diaminobenzidine, and counterstained with hematoxylin. The slides stained with anti-PAI-1 and anti-TGF-β1 antibodies were photographed, and positive staining was quantified from two random fields of view from twelve biological replicates per group at 10× magnification.

### 2.6. Immunofluorescence

For immunofluorescence, the slides were deparaffinized, rehydrated, soaked in 10 mM sodium citrate buffer, and maintained at 95–99 °C for 10 min. After 30 min of cooling, the slides were washed in distilled water three times for 5 min each and then washed in PBS another three times for 5 min each. The slides were blocked with blocking solution for 1 h at room temperature, and then the primary antibody, including an anti-Vimentin antibody (cat# sc-6260, Santa Cruz, Dallas, TX, USA) and an anti-Vinculin antibody (cat# ab129002, Abcam, Cambridge, UK), was added. The slides were incubated overnight at 4 °C. After washing with PBS three times for 5 min each, the secondary antibody was added, followed by incubation for 1 h at room temperature in the dark. The nuclei were stained with 4′,6-Diamidino-2-phenylindole. The immunofluorescence images of all slides were obtained using a confocal laser scanning microscope (LSM 710, Carl Zeiss, DE, Jena, Germany), available at the Soonchunhyang Biomedical Research Core Facility of the Korea Basic Science Institute (KBSI), and immunofluorescence intensity was quantified from two random fields of view from twelve biological replicates per group at the 10× magnification using ImageJ (National Institutes of Health, Bethesda, MD, USA).

### 2.7. Quantitative Reverse Transcription Polymerase Chain Reaction (qRT-PCR) Analysis

For the RNA isolation, the Achilles tendons were harvested along with the surrounding soft tissue and ground to a fine powder in a pre-chilled mortar and pestle with liquid nitrogen. The total RNA was isolated using QIAzol (Qiagen, Germantown, MD, USA) according to the manufacturer’s protocol. Aliquots of 1 μg RNA were reverse-transcribed using a SensiFAST cDNA Synthesis Kit (Bioline, Taunton, MA, USA), according to the manufacturer’s protocol. Briefly, the RNA was denatured at 85 °C for 5 min and 1 μL of reverse transcriptase was added to the final reaction solution (20 μL). The qRT-PCR was performed with 8.4 μL cDNA using a SensiFAST SYBR Hi-ROX kit (Bioline, Taunton, MA, USA) and the specific primers for the rats were TGF-β1, TGF-β2, and a collagen type 1 alpha 1 chain (COL1A1) using a StepOnePlus™ Real-Time PCR System (Applied Biosystems, Foster City, CA, USA), available at the Soonchunhyang Biomedical Research Core-facility of Korea Basic Science Institute (KBSI). The expression levels of the genes of interest were normalized to the expression of glyceraldehyde-3-phosphate dehydrogenase (GAPDH), and ΔCt values were determined as follows: Ct^target^—Ct^GAPDH^. Relative fold changes were calculated using the 2^−ΔΔCt^ method [23]. The PCR primers used are summarized in Table 2.

### 2.8. Statistical Analysis

Statistical analyses were performed using SPSS version 20.0 (SPSS Inc., Chicago, IL, USA). A two-sample *t*-test was used to compare results between the two groups. Data are presented as the mean ± standard error of the mean (SEM) of twelve biological replicates for each group. In all analyses, a *p* value of less than 0.05 was considered to indicate statistical significance.

## 3. Results

### 3.1. Macroscopic and Histological Evaluation

**Figure 2:**
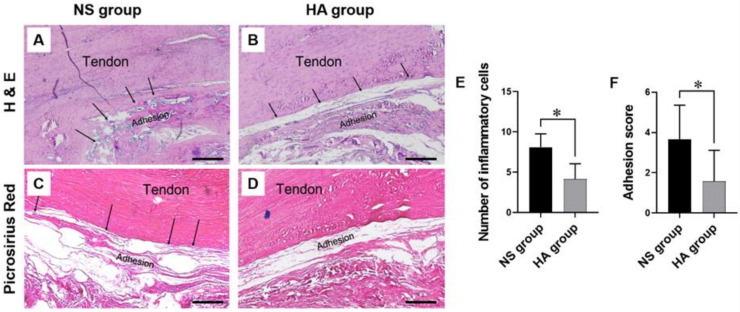
Representative images and analysis of H&E and picrosirius red staining at 10 weeks after surgery. (**A**,**C**) H&E and picrosirius red staining in the normal saline (NS) control group: there is obvious adhesion with moderate density between the repaired tendon and the surrounding soft tissue. The adhesion fibers (arrow) connecting soft tissue to the tendon surface are observed. Scale bar = 100 μm. (**B**,**D**) H&E and picrosirius red staining in the hyaluronic acid (HA) group: a few loose adhesions are observed between the repaired tendon and the sheath, and peritendinous adhesions are mild. The adhesion fibers connecting the subcutaneous tissue and the tendon surface are not observed. Scale bar = 100 μm. (**E**) The mean numbers of inflammatory cells in three serial high-power fields are 8.13 ± 1.65 in the NS control group, and 4.17 ± 1.90 in the HA group (* *p* < 0.05). (*n* = 12 for each group). (**F**) The adhesion score is significantly lower in the HA group (1.58 ± 1.52) than in the control group (3.66 ± 1.68) (* *p* < 0.05). Values represent the means ± standard error of the mean (SEM). (*n* = 12 for each group).

All operations were successful, and all wounds healed well, without complications such as infection or wound disruption. At 10 weeks following surgery, the Achilles tendon tissues were examined using H&E and picrosirius red staining to evaluate the degree of adhesion and tendon recovery after tenorrhaphy (Figure 2A–D). The H&E staining revealed a fine gap between the repaired tendon and the surrounding tissue in the HA group (indicated using black arrows, Figure 2A,B). In contrast, dense collagen fibers were deposited between the repaired tendon and the surrounding tissue in the NS group. The mean number of infiltrating inflammatory cells was significantly different between the NS and HA groups (8.13 ± 1.65 vs. 4.17 ± 1.90, respectively; * *p* < 0.05) (Figure 2E). Picrosirius red staining further confirmed the accumulation of dense and irregular collagen fibers between the repaired tendon and the surrounding tissue in the NS group. In contrast, only a few loose collagen fibers were observed between the repaired tendon and the surrounding tissue in the HA group (Figure 2C,D). The severity of tendon adhesion was scored according to the criteria described in Table 1. In the HA group, no adhesion formation was observed in 33.3% of tendons, slight adhesion formation in 41.8% of tendons, and moderate adhesion formation in 24.9% of tendons. In contrast, in the NS group, only 25% of tendons showed slight adhesion formation, 41.8% of tendons showed moderate adhesion formation, and 33.3% of tendons showed severe adhesion. As shown in Figure 2F, the adhesion score of the HA group was significantly lower than that of the NS group (3.5 vs. 2.11, respectively; * *p* < 0.05).

### 3.2. Immunohistochemistry

**Figure 3:**
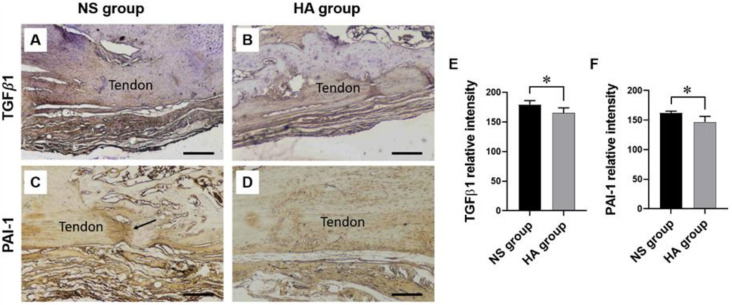
Representative images of transforming growth factor-β1 (TGF-β1) and plasminogen activator inhibitor (PAI-1) immunohistochemical staining and analyses at 10 weeks after surgery. (**A**) TGF-β1 staining in the NS group: dense and irregular collagen fibers are observed between the repaired tendon and the surrounding soft tissue. The collagen fibers forming adhesions and connecting soft tissue to the tendon surface are observed. Scale bar = 100 μm. (**B**) TGF-β1 staining in the HA (experimental) group: a few loose collagen fibers are observed between the repaired tendon and the surrounding tissue. The adhesion fibers have a more regular pattern in the HA group than in the NS group. Scale bar = 100 μm. (**C**) PAI-1 staining in NS group: high expression is observed in the repaired tendon (arrow), and the surrounding adhesion tissue consists of collagen fibers and extracellular matrix. Scale bar = 100 μm. (**D**) PAI-1 staining in the HA group: significantly lower PAI-1 expression is observed in the HA group than in the NS group. Scale bar = 100 μm. (**E**,**F**) Staining results are quantified using ImageJ. The y-axis indicates the mean intensity of staining. Significantly lower (**E**) TGF-β1 and (**F**) PAI-1 expression is observed in the HA group than in the NS group (* *p* < 0.05). Values represent the means ± standard error of the mean (SEM). (*n* = 12 for each group).

Immunohistochemical analysis revealed higher TGF-β1 and PAI-1 expression in the repaired tendon and the surrounding adhesion tissue of the NS group, which exhibited excessive collagen fiber and extracellular matrix (ECM) deposition, than in those of the HA group (Figure 3A–D). The mean intensity of TGF-β1 staining was significantly lower in the HA group than in the NS group (165.04 ± 8.75 vs. 179.58 ± 6.58, respectively; * *p* < 0.05) (Figure 3E). Similarly, the PAI-1 staining intensity was lower in the HA group than in the NS group (146.97 ± 9.26 vs. 162.22 ± 2.91, respectively; * *p* < 0.05) (Figure 3F).

### 3.3. Immunofluorescence

**Figure 4:**
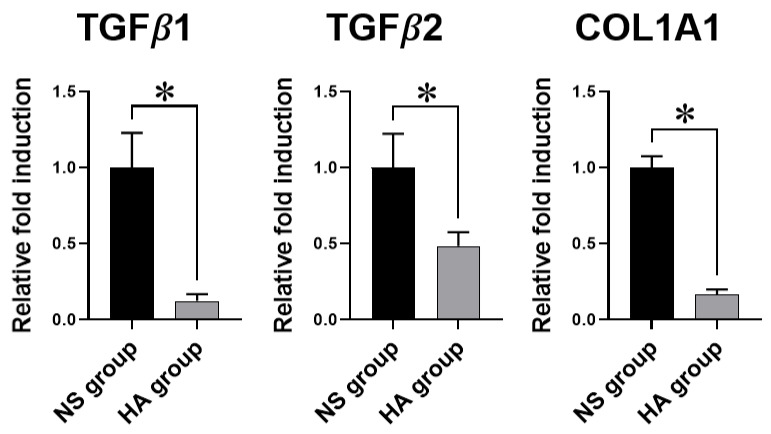
Quantitative polymerase chain reaction (PCR) analysis of fibrosis-associated genes TGF-β1, TGF-β2, and collagen type 1 α1 chain (COL1A1). All gene expression levels are normalized to the levels of the NS group. Glyceraldehyde-3-phosphate dehydrogenase (GAPDH) is used as the reference gene. TGF-β1, TGF-β2, and COL1A1 expression is significantly lower in the HA group than in the NS group (all, * *p* < 0.05). Values represent the means ± standard error of the mean (SEM). (*n* = 12 for each group).

**Figure 5:**
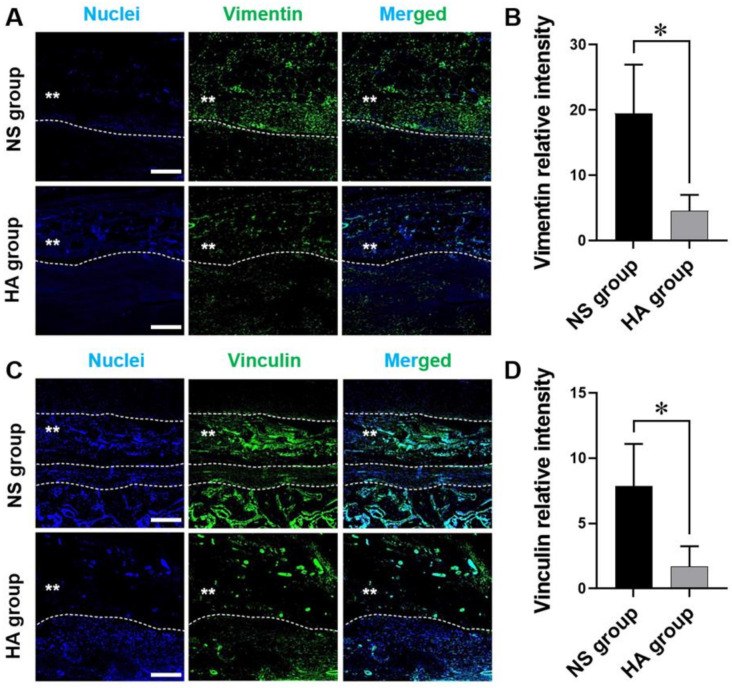
Immunofluorescence staining of vimentin and vinculin and image analyses at 10 weeks after surgery. (**A**,**C**) Immunofluorescence staining of vimentin (**A**, green), vinculin (**C**, green), and cell nuclei (blue); merged images are shown as separate channels. The dashed line indicates the boundary between the tendon and peritendinous adhesion tissue. Scale bar = 200 μm. (**B**,**D**) Immunofluorescence staining is quantified using ImageJ. The y-axis shows the mean intensity of immunofluorescence staining. The fluorescence intensities of vimentin (**B**) and vinculin (**D**) are significantly lower in the HA group than in the NS group (both, * *p* < 0.05). Values represent the means ± standard error of the mean (SEM). (*n* = 12 for each group).

To evaluate the effect of HA on tendon adhesion, we assessed the distribution and degree of vimentin and vinculin expression in both groups. As shown in Figure 4, the mean intensities of vimentin in the NS and HA groups were 19.50 ± 7.47 and 4.59 ± 2.41, respectively, whereas the mean intensities of vinculin were 7.86 ± 3.25 and 1.71 ± 1.55, respectively. Significantly lower levels of vimentin and vinculin protein expression were observed in the HA group than in the NS group (both * *p* < 0.05) (Figure 5).

### 3.4. qRT-PCR Analysis

To further corroborate the immunofluorescence analysis results for vimentin and vinculin, the mRNA expression of TGF-β1 and TGF-β2 in the HA group was as low as 12.3% and 48.5%, respectively, relative to that in the NS group. In addition, the mRNA expression of COL1A1 in the HA group was 16.6% relative to that in the NS group. These differences were all significant (* *p* < 0.05), suggesting that HA injection could inhibit the excess production and adhesion of fibrotic ECM to the surrounding tissue (Figure 4).

## 4. Discussion

The primary aims of tendon repair surgery are to recover the physiologically continuous microstructure of the tendon and to increase tendon mechanical strength during mobilization [24,25]. The major complication that impedes these objectives is the formation of tendon adhesion within the surrounding soft tissue [26]. Although clinical outcomes have improved considerably with recent advances in tendon repair and rehabilitation techniques, there is an ongoing effort to develop novel methods, including the use of anti-adhesive agents, to minimize tendon adhesion during spontaneous healing, thus preventing functionally poor regeneration after surgery [27].

Tendon healing progresses via three stages: tissue inflammation, cell proliferation, and remodeling of the ECM [9,10]. Among these stages, ECM remodeling is considered the most important stage for the regulation of adhesion formation. It begins approximately 6–8 weeks after injury and is characterized by decreased cellularity, reduced matrix synthesis, decreased type III collagen production, and increased type I collagen synthesis [28]. During ECM remodeling, adhesion formation occurs through extrinsic and intrinsic pathways [29,30,31]. During extrinsic healing of tendons, fibroblasts and inflammatory cells invade the healing site from the surrounding tissue and promote tendon tissue repair and regeneration. During intrinsic repair, cells migrate from the endotenon and epitenon into the site of injury and proliferate. The early stages of adhesion formation usually involve the extrinsic pathway, whereas intrinsic repair normally occurs during the later stage. Therefore, adhesion is considered an indispensable by-product of the tendon-healing mechanism, and understanding these pathways is essential for preventing excessive adhesion formation during remodeling.

HA is a disaccharide comprising alternating β-1,4-D-glucuronic acid and β-1,3-N-acetyl-D-glucosamine residues [12]. It is a ubiquitous glycosaminoglycan found in almost all tissues and has important biological roles, including its function as a lubricant in the intra-articular joint space and its involvement in the regulation of vessel permeability [27]. Previous studies demonstrated that the use of HA decreased adhesion formation in vivo [32,33,34]. However, the mechanism of action of HA in preventing tendon adhesion remains elusive. There are two main hypotheses regarding the mechanism underlying HA’s effects. The first is that HA acts as a physical barrier around the tendon repair site, shielding the tendon from other physiological reactions that may induce excessive scar formation [19]. The second hypothesis is that the principal effect of HA is either pharmacological or physiological, resulting in decreased ECM formation by the inhibition of the activity of mononuclear phagocytes and lymphocytes [18].

Therefore, to obtain a better understanding of HA’s mechanism of action, we investigated the effects of HA injection on adhesion formation in the Achilles tendons of rats. To analyze the degree of adhesion and tendon recovery, tendons were examined by H&E and picrosirius red staining to determine the distribution and density of collagen fibers after tendon repair surgery. The results indicated almost no adhesion formation in the HA group, which was evidenced by the lesser collagen deposition observed between the tendon and the surrounding tissue, compared with that in the control group. Denser aggregates of collagen fibers were formed and attached to each other in the tendons of the NS group than in those of the HA group. These observations clearly indicated that HA injection exhibited a significant anti-adhesive effect. In addition, there were significantly higher numbers of infiltrating inflammatory cells at the tenorrhaphy site in the NS group than in the HA group, suggesting that HA could alleviate excessive inflammation related to adhesion formation.

TGF-β1 was previously shown to stimulate the migration and proliferation of fibroblasts, promoting their differentiation into myofibroblasts, which produce excessive ECM and subsequently activate PAI-1, a serine protease inhibitor, suppressing tissue and urokinase plasminogen activator irreversibly [35,36]. The activation of TGF-β1 directly upregulates PAI-1 activity, and, therefore, both are abundant in healing and in scar tissue following tendon injury [36]. Thus, numerous research groups have investigated the potential of TGF-β1 and PAI-1 as therapeutic targets for preventing adhesion formation. In the current study, specimens were immunohistochemically evaluated for TGF-β1 and PAI-1 expression in the tenorrhaphy sites. In accordance with the findings of previous reports, our findings indicated that HA injection significantly downregulated both TGF-β1 and PAI-1 expression compared to the control treatment [36,37].

Adhesions and scar tissue are also characterized by the excessive accumulation of collagen and other ECM components [38]. Among ECM components, collagen plays a key role in tendon healing and contributes to adhesion formation. During the remodeling phase of tendon regeneration, type I collagen fibers are organized along the tendon axis and are responsible for the reinforcement of the tendon’s mechanical strength [9,28]. However, the excessive production of type I collagen triggered by TGF-β1 during remodeling could lead to fibrotic tissue and adhesion formation within the tendon and the surrounding tissue. In addition, TGF-β2 has been implicated in the early stages of tendon development as well as tendon ECM synthesis such as type I/III collagen and elastin at the late stages [39,40]. Similarly, it has been reported that the activation of TGF-β2 can promote the synthesis of type I/III collagen in tendon progenitor cells [41,42]. Thus, regulation of collagen synthesis is key to preventing adhesion formation after surgery. In the present study, significant downregulation of TGF-β1, TGF-β2, and COL1A1 gene expression was observed in the HA group, suggesting that HA could reduce adhesion by regulating TGF-β1 and PAI-1 expression during the remodeling phase.

Because the formation of tendon adhesions is closely associated with the dynamic interaction between tenocytes and their surrounding ECM, understanding focal adhesions and cell–ECM interactions is also very important [43,44]. Vinculin, a 116-kDa protein, is highly enriched in regions where cells make contact with each other [45] and is known as a major regulator of cell–matrix adhesion via its interaction with specific phospholipids in adhesion complexes [46]. In addition, vimentin is a 57-kDa type III intermediate filament that connects to focal adhesions through filamin A [47,48]. Excessive focal adhesions could subsequently induce the formation of tendon adhesions [49]. To investigate the effects of HA on focal adhesion complex formation, we assessed vinculin and vimentin expression. Focal adhesions were fewer in number and smaller in size, and the vinculin and vimentin staining intensities were lower in the HA group than in the NS group (Figure 5). Similarly, Chen et al. have recently developed HA-based multi-functional nanofibrous membranes (NFMs) loaded with ibuprofen to mimic the anti-adhesive function of the native tendon sheath, as well as to induce anti-inflammatory responses at the tenorrhaphy sites [50]. They successfully demonstrated that ibuprofen-loaded HA-NFM could minimize the cell attachment, as evidenced by the minimal expression of vinculin and restricted cytoskeleton organization, resulting in the prevention of cell penetration and tendon adhesion formation during tendon healing. Taken together, these results indicate that HA could reduce focal adhesion formation and cell–matrix adhesion, thereby suppressing tendon adhesion formation.

## 5. Conclusions

The results of the present study elucidate the effects of HA injection on the extrinsic and intrinsic pathways of tendon adhesion formation during the healing phase. Our results highlight that the injection of HA could reduce adhesion formation by significantly ameliorating inflammatory-associated reactions, as revealed by microscopical and histological evaluation, in addition to the downregulation of TGF-β1, TGF-β2, and COL1A1 expression.

## Figures and Tables

**Table 1:** Criteria for the histological evaluation of peritendinous adhesions.

Score	Features of Adhesion
**Quantity**	
0	No apparent adhesions
1	A number of scattered filaments
2	A large number of filaments
3	Countless filaments
**Quality**	
0	No apparent adhesions
1	Regular, elongated, fine filamentous
2	Irregular, mixed, shortened, filamentous
3	Dense, not filamentous
**Grading of Adhesions**	
0	None
1–2	Slight
3–4	Moderate
5–6	Severe

**Table 2:** Primer sequences for quantitative reverse transcription polymerase chain reaction.

Accession Number	Gene	Forward Primer	Reverse Primer	Amplicon Size
NM_021578.2	TGF-β1	5′-GCCTGAGTGGCTGTCTTTTGA-3′	5′-GGCTGATCCCGTTGATTTCCA-3′	146 bp
NM_031131.2	TGF-β2	5′-CATCCCGCCCACTTTCTACAG-3′	5′-CACTCTGGCTTTGGGGTTTTG-3′	133 bp
NM_053304.1	COL1A1	5′-CCCGAACCCCAAGGAAAAGAA-3′	5′-TAGGCTACGCTGTTCTTGCAG-3′	183 bp
NM_017008.4	GAPDH	5′-TCACCACCATGGAGAAGGC-3′	5′-GCTAAGCAGTTGGTGGTGCA-3′	169 bp

## Data Availability

The data presented in this study are available on request from the corresponding authors.

## Abbreviations

TGF-β1	transforming growth factor-beta 1
TGF-β2	transforming growth factor-beta 2
COL1A1	collagen type 1 alpha 1 chain
GAPDH	glyceraldehyde-3-phosphate dehydrogenase
